# Reporting and Performance of Hepatocellular Carcinoma Risk Prediction Models: Based on TRIPOD Statement and Meta-Analysis

**DOI:** 10.1155/2021/9996358

**Published:** 2021-08-02

**Authors:** Liuqing Yang, Qiang Wang, Tingting Cui, Jinxin Huang, Hui Jin

**Affiliations:** ^1^Department of Epidemiology and Health Statistics, School of Public Health, Southeast University, Nanjing, China; ^2^Key Laboratory of Environmental Medicine Engineering, Ministry of Education, School of Public Health, Southeast University, Nanjing, China

## Abstract

**Background:**

The performance of risk prediction models for hepatocellular carcinoma (HCC) in patients with chronic hepatitis B (CHB) was uncertain. The aim of the study was to critically evaluate the reports of transparent and external validation performances of these prediction models based on system review and meta-analysis.

**Methods:**

A systematic search of the Web of Science and PubMed was performed for studies published until October 17, 2020. The transparent reporting of a multivariable prediction model for the individual prognosis or diagnosis (TRIPOD) tool was used to critically evaluate the quality of external validation reports for six models (CU-HCC, GAG-HCC, PAGE-B, mPAGE-B, REACH-B, and mREACH-B). The area under the receiver operator characteristic curve (AUC) values was to estimate the pooled external validating performance based on meta-analysis. Subgroup analysis and metaregression were also performed to explore heterogeneity.

**Results:**

Our meta-analysis included 22 studies published between 2011 and 2020. The compliance of the included studies to TRIPOD ranged from 59% to 90% (median, 74%; interquartile range (IQR), 70%, 79%). The AUC values of the six models ranged from 0.715 to 0.778. In the antiviral therapy subgroups, the AUC values of mREACH-B, GAG-HCC, and mPAGE-B were 0.785, 0.760, and 0.778, respectively. In the cirrhosis subgroup, all models had poor discrimination performance (AUC < 0.7).

**Conclusions:**

A full report of calibration and handling of missing values would contribute to a greater improvement in the quality of external validation reports for CHB-related HCC risk prediction. It was necessary to develop a specific HCC risk prediction model for patients with cirrhosis.

## 1. Introduction

Hepatitis B virus (HBV) was one of the crucial causes of hepatocellular carcinoma (HCC), accounting for approximately 50% of all cases of HCC [1, 2]. It was well known that chronic hepatitis B (CHB) patients were primarily concentrated in East Asia, South Asia, and sub-Saharan Africa [3, 4]. It was noteworthy that the incidence of CHB-related HCC was increasing, especially in Western countries [5]. If HCC can be diagnosed earlier in the monitoring process, the diversity of treatment options and the probability of cure would be higher and the long-term prognosis would certainly be improved. Therefore, it was critical to identify and closely monitor high-risk patients. This would enable those patients to receive timely intervention.

Risk prediction models had a long history in being used for predicting the incidence of HCC in patients with CHB. The highly popular and recognized models were as follows: CU-HCC, GAG-HCC, PAGE-B, mPAGE-B, REACH-B, and mREACH-B [6, 7]. The clinical application of these models can assist the prognosis and decision-making of patients. Existing models have been developed in different settings, such as untreated patients, receiving antiviral therapy patients, and mixed patients. However, published guidelines seldom provided standard methods to assess HCC risk prediction in patients with CHB [8–10]. Additionally, some issues had been found in the risk prediction models, such as use of nonstandard methodology, scarce external validation, and excessive reduction of discrimination in new cohorts [11]. It was uncertain whether the above problems would occur in the HCC prediction models. The complete reporting is conducive to research replication and evaluating its applicability to other individuals. Therefore, high-quality reports on prediction models are essential. In 2015, multiple journals simultaneously published a study on how to improve the quality of predictive model research reports, that is, transparent reporting of individual prognosis or diagnostic multivariate predictive model (TRIPOD) statement [12].

The purpose of this study was to systematically evaluate the reports of transparent and completeness of these external validation and then analyze the prediction performance of the models based on meta-analysis.

## 2. Methods

This study was performed in accordance with the Preferred Reporting Items for Systematic Reviews and Meta‐Analysis (PRISMA). No approval statement was required for this systematic review and meta-analysis because the data included in our study had been published previously. Patients/ the public were not involved in the design, implementation, reporting, or dissemination plan of the study.

### 2.1. Search Strategy

A search was conducted on Web of Science and PubMed databases until October 17, 2020, with no language and publication dates restrictions. Details of search strategy were provided in Supplementary Material (the part of “Search Strategy”). We also checked reviews in this field and references of the original articles to identify whether there were any missed studies.

### 2.2. Selection Criteria

All studies that used CU-HCC, GAG-HCC, REACH-B, mREACH-B, PAGE-B, and mPAGE-B models to predict the risk of HCC in CHB patients were included in our study. The exclusion criteria were as follows: (1) nonhuman subjects, (2) research aiming at specific populations, such as children and patients with other serious diseases (HCV or HIV, etc.), (3) original studies on development of HCC prediction models (only described developmental research or internal verification), and (4) updates on HCC prediction models (without the external verification of the original model).

### 2.3. Data Extraction

Two investigators (LQY, TTC) independently reviewed the titles and abstracts of all extracted articles. The following data were extracted from these studies: (1) the first author's name, (2) year of publication, (3) study interval, (4) study region, (5) outcome to be predicted, (6) study race, (7) study setting, (8) sample size, (9) type of antiviral treatment received, and (10) the discrimination and calibration of prediction models, including area under the receiver operator characteristic curve (AUC) and observed to expected ratio (O:E ratio), and its standard error (SE) or 95% confidence interval (CI). For external validation of different existing models, information was extracted separately.

The TRIPOD statement was used to evaluate the quality of the included studies (http://www.tripod-statement.org/). Specific information on the method of TRIPOD assessment is provided in the Supplementary Materials section (the part of “Tripod Statement”). The Prediction Model Risk of Bias Assessment Tool (PROBAST) was used to assess bias (participants, predictors, outcome, and analysis) in the included studies. Specific evaluation forms used were available on the official website (http://www.probast.org).

### 2.4. Statistical Analyses

We estimated the values of discrimination and calibration with their SE or 95% CI on the logit scale and pooled the statistical values and SE in the meta-analysis [13]. For studies that did not provide SE values, we calculated them from the reported upper and lower limits of the CI or from the reported *p* value [13, 14]. The *Z* Test was used to compare the AUC values between models. The *I*^2^ statistic was used to assess the heterogeneity among the studies. When *I*^2^ statistic >50% was considered as moderate heterogeneity, the random effect model was used for analysis; otherwise, the fixed effect model was used for analysis (http://handbook.cochrane.org).

Subgroup analysis and metaregression were performed to explore potential sources of heterogeneity. The subgroups were stratified by presence of hepatic cirrhosis (cirrhosis or no-cirrhosis), administration of antiviral therapy (received or no-received), and follow-up time. All statistical analyses were performed using Stata14.

## 3. Results

**Figure 1:**
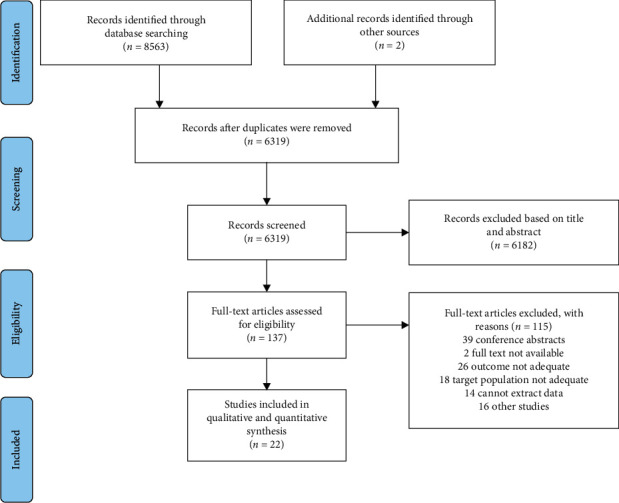
Flowchart of literature search.

After screening, a total of 22 publications were included in our analysis (see Figure 1). From these publications, 58 models were evaluated as follows: CU-HCC (*n* = 14), GAG-HCC (*n* = 11), PAGE-B (*n* = 7), mPAGE-B (*n* = 3), REACH-B (*n* = 19), and mREACH-B (*n* = 4).

### 3.1. Primary Information

Patients' data of fifteen studies were from Asia (including China, Japan, and South Korea), five studies from Europe (including Spain, the Netherlands, Italy, and Greece), and North America (Canada) and the United States and the Asia-Pacific region had one each. The ethnicity of the study population included Asians, Caucasians, and Africans. Most of them received antiviral therapy during the follow-up period, including nucleotides, interferon, and lamivudine. The setting of all included studies was limited to hospitals. The median sample size was 1000 (interquartile range (IQR): 557, 1505). Detailed information is shown in Table 1.

### 3.2. Reporting Completeness per Model in TRIPOD

Overall, publication compliance with TRIPOD ranged from 59% to 90%, with a median of 74% (IQR: 70%, 79%). The compliance of each model was as follows, CU-HCC (59%–81%; median, 71%; IQR, 68%, 77%), GAG-HCC (59%–81%; median, 72.5%; IQR, 68%, 79%), PAGE-B (71%–79%; median, 75%; IQR, 73%, 79%), REACH-B (59%–90%; median, 74%; IQR, 69%, 79%), and mREACH-B (70%–81%; median, 76.5%; IQR, 71%, 81%). The results were shown in Supplementary Material (the part of “Reporting Completeness per Model and Items in Tripod”).

### 3.3. Reporting Completeness per TRIPOD Item

There were 31 items in the TRIPOD for external validation reports. Completeness of 23 items reached 75% or more, up to a maximum of 100%; 6 items was below 25%, with a minimum of 0%.

In the “Title and Abstract” domain, seven models (41.18%) were able to meet the requirements of item 1 with complete titles. The abstract reporting of item 2 was not complete for most, with only one model (1.96%) meeting all the requirements. However, the background information and study objectives requirements of items 3-4 were completely fulfilled in all models. In the “Methods” domain, the item 9 requirement, description of missing data was incomplete in most of the models, with only seven models describing ways to deal with the missing data in detail, accounting for 13.73%. Few studies (1.96%) used calibration as a measure to evaluate the performance of the prediction models. In the “Results” domain, seven models (13.73%) compared the distribution of important variables in the study with data from original developmental studies. Compared with discrimination, just one model provided the calibration, so the compliance for item 6 was low (1.96%). None of the models provided details of missing data of the predictors and predictive outcomes mentioned in item 13b. In the “Discussion” and “Other Information” domains, reports of almost all models fulfilled requirements of items 18-22, with compliance of over 80%. The detailed results were shown in Supplementary Materials (the part of “Reporting Completeness per Model and Item in Tripod”).

### 3.4. Meta-Analysis

In meta-analysis, we screened 17 studies (42 external validation on models) of the included studies, from which AUC values could be extracted. Detailed data has been included in the Supplementary Material (see Table S1). Calibration could not be used in the meta-analysis because of the small number of extracted O:E ratio values. As shown in Table 2, the AUC values of the models ranged from 0.715 to 0.778. The performance seen in REACH-B (0.715; 95% CI, 0.673, 0.754) was lower than that in mPAGE-B (0.778; 95% CI, 0.749, 0.804) and GAG-HCC (0.775; 95% CI, 0.742, 0.804) (*p* < 0.05).

### 3.5. Subgroup Analysis

The results of all subgroup analyses are shown in Table 3. In the antiviral therapy subgroup, the AUC values of mREACH-B (AUC, 0.785; 95% CI, 0.750, 0.817) were relatively higher than those of CU-HCC (AUC, 0.731; 95% CI, 0.705, 0.756) and REACH-B (AUC, 0.639; 95% CI, 0.612, 0.666) (*p* < 0.05). The REACH-B was relatively lower than others (*p* < 0.05). In the no-antiviral therapy subgroup, data for PAGE-B and mPAGE-B were not included because both models were developed in cohorts receiving antiviral therapy. Among the remaining four models in this subgroup, we found that all of models had similar AUC values. In subgroup comparison between antiviral and no-antiviral therapies, the discrimination performance of REACH-B in no-antiviral therapy subgroup was found to be higher than the other (*p* < 0.05). The difference was statistically significant.

All models showed poor discrimination performance (<0.7) in the cirrhosis subgroup. It was noteworthy that the AUC value in CU-HCC was only 0.582 (95% CI, 0.522, 0.641). Moreover, mREACH-B showed similar or higher discrimination power among the three models (AUC, 0.688; 95% CI, 0.653, 0.721). In the no-cirrhosis subgroup, both mREACH-B (AUC, 0.785; 95% CI, 0.750, 0.817) and REACH-B (AUC, 0.774; 95% CI, 0.721, 0.820) showed better performance than in the cirrhosis subgroup (*p* < 0.05).

### 3.6. Risk of Bias and Applicability

The assessment based on the PROBAST was shown in Supplementary Figure S1. Regarding the part of bias (see Figure S1(a)), more than half of the models were reported to be of low risk (58.8%), high-risk models accounted for 37.3%, and no information was 4%. In the participant domain, the distribution of some externally validation was different from that of the original model in selection of the study population. In the analysis domain, almost all models had the issue of not providing a method to deal with missing data. In addition, ignoring the complexity of the data, the method of processing continuous variables and classified variables was not clear. Regarding the part of applicability (see Figure S1(b)), most prediction models were considered to be of low risk, accounting for 94%. Three models were eventually evaluated to be lacking relevant information since the settings for participants or the definition of predictors was not provided. No model was evaluated to be of high risk.

### 3.7. Metaregression Analysis

Table 4 shows that heterogeneity was not explained well by metaregression. In the REACH-B model, heterogeneity may be associated with cirrhosis and antiviral therapy. In the mREACH-B model, cirrhosis may be the cause of heterogeneity.

## 4. Discussion

We reviewed 22 studies, all of which were aimed at external validation of the six risk models used for prediction of CHB-related HCC risk. Using the TRIPOD tool, we found that the compliance of these studies to items of TRIPOD was at a medium level. Meta-analysis demonstrated that mREACH-B exhibited comparable or superior performance to other models both overall and in subgroups, while the REACH-B was low.

Overall, in TRIPOD, 59% to 90% of compliance was seen in the included studies, with a median of 74%. Almost all inclusion models could provide a detailed description of the theoretical basis and the data source used in the studies. There were a few common issues in all models. Firstly, the words “validation” or “validating” did not appear in the title of the article, which may not have a great impact on the quality of the article; however, it created a hindrance to the researcher in identification of the articles in a search for published literature in this field. Secondly, almost all models failed to explain ways to deal with missing data; though, in the illustrative articles, complete case analysis, instead of multiple imputation or single imputation, was used to fill the missing data. This may lead to loss of a large amount of data, thus finally having a direct impact on the predictive performance of the model. Thirdly, the data on model calibration were not provided in all studies, which limited our meta-analysis by pooling O:E ratio values. Hence, we could not assess the calibration performance of the models. Fourthly, most of the studies did not compare the characteristics of participants involved in external validation with those of the derived cohort. In general, the TRIPOD compliance was at a medium level. However, compared with the data published by the TRIPOD team in 2018 (median, 43%; IQR, 37%, 54%), a great improvement had been achieved [15].

To date, there were very few reviews on external validation of the established HCC prediction models. A few studies were committed to developing new prediction models, but they were not validated externally. In addition, most studies describe the characteristics of participants and divide them into different subgroups. All of the above factors may result in the discrimination and calibration performance of the newly established prediction models being too optimistic to meet real-world requirements in patients with CHB.

Better or even excellent prediction performance that had been shown in the original data set (all above 0.8) was not reproduced in our study [6, 16–20]. It was inferred that the original study on these models may demonstrate the issue of overfitting. Both Hawkins [21] and Babyak [22] believed that overfitting would greatly reduce the scientific value of research and create great uncertainty. Additionally, we can see that the discrimination performance of mPAGE-B and GAG-HCC was higher than that of REACH-B. In external validation of the original model development study concerning the two models, we also found that the two models maintained the highest discrimination performance. Additionally, Lee et al. confirmed that the performance of mPAGE-B was similar to that of GAG-HCC and significantly higher than that of CU-HCC and REACH-B [23]. Wong et al. [24] and Arends et al. [25] also reported that the prediction performance of GAG-HCC was higher than that of REACH-B. We believed that this may be due to the large heterogeneity between participants in different external validation cohorts.

Our study revealed that the discrimination power of the model was similar in the no-antiviral subgroup, but there was a difference in the antiviral subgroup. This indicated that the value of serum HBV-DNA levels was diminished because of the wide use of effective antiviral drugs. This also confirmed that the model with HBV-DNA as a predictor should be used prudently when predicting the risk of HCC in patients who have been treated with antiviral therapy. Lee et al. reported that, in the era of antiviral therapy, if the interpretation to virus was not considered, the exciting prediction model would make the outcomes detached from the reality [26]. Liu et al. also verified this view, in the point of biology, that RNA might be a more direct marker of cccDNA than the suppressed HBV-DNA due to halted pgRNA reverse transcription [27]. Besides, we found that the discrimination of mPAGE-B was comparable to that of mREACH-B both overall and in the antiviral therapy subgroup, which was significantly higher than that of the other models. This suggested that mPAGE-B was a robust prediction model and was universally applicable in clinical practice. This may rely on the predictor of serum albumin in this model. Kim et al. suggested that serum albumin was an independent risk factor for HCC [16]. Jeon et al. [28] and Papateodoridis et al. [29] also recognized the value of serum albumin as a predictor. Therefore, we inferred that serum albumin as a clinical indicator may have a high practical significance in evaluating the development of CHB-related HCC. However, few studies have assessed the incidence of HCC in this subgroup; hence, larger cohorts and more data were needed for confirmation. Moreover, the predictive performance of the REACH-B model was lower both overall and in antiviral subgroups. This contradicted the findings of Magalhaes et al. [30] and Chen et al. [31], both of whom reported that the REACH-B model maintained its predictive power in the background of receiving antiviral therapy. This may be because of the insufficient number of studies included in this paper, and more data on the predictive performance of the REACH-B model were needed for meta-analysis research. Another reason may be that REACH-B model was initially established in patients without cirrhosis, while the external validation cohorts contained cirrhotic patients, which may also lead to the decline of the model's discrimination performance.

Several studies have confirmed that effective antiviral therapy can reduce the risk of HCC by inhibiting the replication of HBV, thus improving the long-term prognosis of patients with CHB [32, 33]. However, existing studies also confirmed that the probability of HCC occurrence was not completely eliminated [34]. The main reason for this was the existence of cirrhosis. Cirrhosis, which was an important risk factor for HCC, cannot be resolved by inhibiting viral replication. In subgroup analysis, mREACH-B showed higher discrimination performance. This may be because the mREACH-B depended on the sensitivity of liver stiffness (LS) values rather than on a simple binary classification of liver cirrhosis to predict outcomes. Studies included in this study diagnosed patients to be with cirrhosis or no-cirrhosis based on imaging or clinical data rather than histological examination. Liver biopsy was seldom used as a screening tool. Therefore, the presence or absence of cirrhosis was highly likely to be misclassified, and patients with early cirrhosis might be missed in these studies, owing to observer differences, etc. Hence, the predictive performance of models that use a binary classification of liver cirrhosis as predictors, such as CU-HCC, would naturally be greatly limited in cirrhosis subgroup. Although serum albumin was included in the CU-HCC score as a predictor, theoretically compensating for the misclassification of cirrhosis by clinical criterion, the predictive performance of CU-HCC was still poor in this subgroup. Paradoxically, Abu et al. concluded that the better predictive ability of CU-HCC was because of the large weight of predictors of liver function in the CU-HCC [35]. However, our study found that these weight advantages were not reflected in the cirrhosis subgroup. Although we just stated that the gold standard for diagnosis of cirrhosis was histological evaluation, it was not routinely feasible as a screening tool in the clinic. Conventional imaging methods such as ultrasonography and computed tomography, which were routinely used, also have been limited to use because of their inherent operator dependence and subjective variance in interpretation of results as well as the limitations in repeated examinations. Therefore, LS was considered a reliable criterion for assessing the degree of liver fibrosis [36, 37]. In our study, the model in which LS value was included as a predictor, the mREACH-B, had relatively high feasibility and accuracy. Jung et al. reported enhanced performance of the REACH-B model when they replaced the predictor, serum HBV-DNA with LS value in the model. This corelated to the finding in our study [38].

In particular, it should be noted that, in the cirrhosis subgroup, poor discrimination performance was observed in each model. This suggested that these models were unable to accurately predict the risk of HCC in CHB patients with cirrhosis. It may be inferred that qualitative values alone cannot provide additional discrimination ability of prediction models in patients with liver cirrhosis. In view of this, relevant decisions were needed for prediction models in this subgroup in the future. For example, we need to see if the predictors may be quantified so that the prediction performance of the model would improve.

Our study had several limitations. First, there have not been enough studies on external validation of prediction models, so only a few studies were included. Second, the participants included in our meta-analysis were all from medical institutions, not a community-based cohort, which increased the probability of participant bias. In the future, studies needed to be conducted on community-based cohort to improve the prediction performance of the models and thus make the models more universally applicable. And primary information about the participants in the included articles has not been well reported, which limited our analysis of the heterogeneity sources of the data. Third, since the O:E ratio data could not be extracted, meta-analysis could not be conducted on calibration. Fourth, due to the lack of data, predictive performance in the subgroup with higher alanine aminotransferase (ALT) levels could not be evaluated for the mREACH-B model. It was known that higher ALT levels may lead to higher mREACH-B scores, thus affecting its accuracy in prediction. Further research was needed to focus on the use of the mREACH-B model in subpopulations with elevated ALT levels.

## 5. Conclusion

In conclusion, the external validation articles of models predicting the risk of HCC in CHB patients were mediumly standardized and needed to be further improved, particularly in the handling of missing values and reporting of model calibration data. From this, journals receiving manuscripts dealing with disease risk prediction models should require authors to complete the TRIPOD tool for achieving transparent and standardized reporting. The mREACH-B showed relatively stable discrimination performance, while REACH-B was not ideal. However, considering the significant differences in the incidence of HCC in no-cirrhotic and cirrhotic patients, it was especially necessary to develop a new HCC risk prediction model for patients with cirrhosis in future researches.

## Figures and Tables

**Table 1:** The basic features of included studies.

	The first author	Year	Study interval	Region	Race	Setting	Model	Antiviral therapy	Sample size
1	Wong, G. L.	2013	2005.12–2012.8	China	Asian	Hospital	CU-HCC, GAG-HCC, and REACH-B	Entecavir	1531
2	Abu-Amara, Mahmoud	2016	—	Canada	Asian, Caucasian, and African	Hospital	CU-HCC, GAG-HCC, and REACH-B	Nucleotide analog, interferon	2105
3	Arends, P.	2015	2005–2013.5	European	Asian, Caucasian	Hospital	CU-HCC, GAG-HCC, and REACH-B	Entecavir	744
4	Lee, Hye Won	2019	2010–2016	Korea	Asian	Hospital	CU-HCC, GAG-HCC, PAGE-B, mPAGE-B, and REACH-B	Entecavir, tenofovir, and lamivudine	1330
5	Jeon, Mi Young	2018	2006–2014	Korea	Asian	Hospital	CU-HCC, REACH-B, and mREACH-B	No-classification	1397
6	Papatheodoridis, George V.	2015	—	Greece, Italy Spain, The Netherlands, and Turkey	Caucasian	Hospital	CU-HCC, GAG-HCC, and REACH-B	Entecavir, tenofovir,	1666
7	Kim, G. A.	2015	1997.1.1–2012.12.31	Korea	Asian	Hospital	CU-HCC	None	829
8	Kim, Ji Hyun	2018	2007.1–2016.6	Korea	Asian	Hospital	CU-HCC, GAG-HCC, PAGE-B, mPAGE-B, and REACH-B	Entecavir, tenofovir	1000
9	Brouwer, W. P.	2017	1985–2012	Rotterdam, The Netherlands	Asian, Caucasian, and African	Hospital	CU-HCC, GAG-HCC, PAGE-B, and REACH-B	No-classification	557
10	Yang, H. I.	2019	1997–2016	American, Asian-Pacific region	Asian American, and Asian	Hospital	CU-HCC, GAG-HCC, PAGE-B, and mPAGE-B, REACH-B	Oral antiviral	2683
11	Jeon, M. Y.	2017	2006.4–2014.12	Korea	Asian	Hospital	CU-HCC, REACH-B, and mREACH-B	No-classification	540
12	Tawada, Akinobu	2016	2000.11–2014.3	Japan	Asian	Hospital	CU-HCC, GAG-HCC	Entecavir, lamivudine	225
13	Jung, K. S.	2015	2006–2011	Korea	Asian	Hospital	CU-HCC, GAG-HCC, REACH-B, and mREACH-B	No-classification	1308
14	Kim, M. N.	2017	2006.8–-2015.1	Korea	Asian	Hospital	CU-HCC, GAG-HCC, PAGE-B, and REACH-B	Entecavir, tenofovir	1092
15	Riveiro-Barciela, M.	2017	2005.4–2015.09	Spain	Caucasian	Hospital	PAGE-B,	Emtricitabine, entecavir, tenofovir, and lamivudine	611
16	Seo, Yeon Seok	2017	2006–2012	Korea	Asian	Hospital	PAGE-B, REACH-B, and mREACH-B	—	1241
17	Magalhaes-Costa, Pedro	2016	2006.1–2014.2	Spain	Asian, Caucasian, and African	Hospital	REACH-B	Entecavir, tenofovir	120
18	Yang, H. I.	2016	—	China	Asian	Hospital	REACH-B	ERADICATE-B (none) CUHK (1/4)	ERADICATE-B (2688) CUHK(426)
19	Chen, T. M.	2013	2006.1–2012.5	China	Asian	Hospital	REACH-B	—	904
20	Chen, W.	2015	2004.10.1–2014.5.1	China	Asian	Hospital	REACH-B	—	627
21	Lee, H. W.	2014	2007.2–2011.1	Korea	Asian	Hospital	REACH-B	Entecavir	192
22	Yang, H. I.^*∗*^	2011	—	China, Korea	Asian	Hospital	REACH-B	None	1505

**Table 2:** The predictive performance of each model in CHB-related HCC patients.

Model	AUC^†^		
	Value	95% CI^‡^	*I*^2^ (%)
CU-HCC	0.732	0.664, 0.791	84.1
GAG-HCC	0.775	0.742, 0.804	54.4
PAGE-B	0.735	0.708, 0.762	12.3
mPAGE-B	0.778	0.749, 0.804	43.7
REACH-B	0.715	0.673, 0.754	81.6
mREACH-B	0.759	0.703, 0.807	62.9

^†^AUC: area under the receiver operator characteristic curve; ^‡^CI: confidence interval.

**Table 3:** Analysis of the predictive performance of each model in subgroups.

Model	Subgroup		AUC^†^	95% CI^#^	*I* ^2^
CU-HCC	Antiviral therapy	Receiving	0.731	0.705, 0.756	0.0%
		Not receiving	0.731	0.689, 0.768	0.0%
	Cirrhosis	Yes	0.582	0.522, 0.641	45.8%
		No	0.672	0.489, 0.815	85.1%

GAG-HCC	Antiviral therapy	Receiving	0.760	0.734, 0.783	1.1%
		Not receiving	0.773	0.681, 0.845	0.0%
	Cirrhosis	Yes	—^§^	—	—
		No	—	—	—

PAGE-B	Antiviral therapy	Receiving	0.741	0.707, 0.772	28.2%
		Not receiving	—	—	—
	Cirrhosis	Yes	—	—	—
		No	—	—	—

mPAGE-B	Antiviral therapy	Receiving	0.778	0.749, 0.804	43.7%
		Not receiving	—	—	—
	Cirrhosis	Yes	—	—	—
		No	—	—	—

REACH-B	Antiviral therapy	Receiving	0.639	0.612, 0.666	0.0%
		Not receiving	0.763	0.744,0.781	37.9%
	Cirrhosis	Yes	0.648	0.613, 0.682	0.0%
		No	0.774	0.721, 0.820	79.6%

mREACH-B	Antiviral therapy	Receiving	0.785	0.750, 0.817	23.4%
		Not receiving	0.789	0.737, 0.833	0.0%
	Cirrhosis	Yes	0.688	0.653, 0.721	0.0%
		No	0.785	0.700, 0.851	65.5%

AUC: area under the receiver operator characteristic curve; CI: confidence interval. The symbol § means that the information cannot be extracted.

**Table 4:** The results of metaregression.

Model	Variable		Coefficient	95% CI^‡^	*t*	*p*
CU-HCC	Cirrhosis	−0.329	−2.145, 1.488	−0.78	0.518	
	Antiviral therapy	0.003	−0.287, 0.293	0.02	0.983	
	Multiple time points	3-year	0.561	−0.398, 1.520	1.25	0.230
		5-year	0.479	−0.447, 1.405	1.11	0.286

GAG-HCC	Cirrhosis	—^‡^	—	—	—	
	Antiviral therapy	−0.078	−0.688, 0.531	−0.31	0.764	
	Multiple time points	3-year	0.307	−0.390, 1.003	1.00	0.345
		5-year	—	—	—	—

PAGE-B	Cirrhosis	—	—	—	—	
	Antiviral therapy	—	—	—	—	
	Multiple time points	3-year	0.0166	−0.319, 0.353	0.13	0.904
		5-year	—	—	—	—

REACH-B	Cirrhosis	−0.611	−1.109, −0.114	−2.71	0.020	
	Antiviral therapy	−0.598	−0.771, −0.424	−7.51	0.000	
	Multiple time points	3-year	0.457	−0.372, 1.286	1.15	0.264
		5-year	0.064	−0.780, 0.907	0.16	0.877

mREACH-B	Cirrhosis	−0.529	−1.016, −0.042	−2.79	0.038	
	Antiviral therapy	−0.020	−0.586, 0.546	−0.11	0.918	
	Multiple time points	3-year	0.211	−0.374, 0.796	0.83	0.431
		5-year	−0.044	−0.663, 0.576	−0.16	0.875

^†^CI: confidence interval. The symbol ^‡^means that the information cannot be extracted.

## Data Availability

The data included in our study had been published previously, and the data generated in this study have been included within the manuscript.

## Supplementary Materials

Supplementary Materials This section includes the domains of Search Strategy, Tripod Statement, and Reporting Completeness per Model and Item in TRIPOD. Table S1: the extracted AUC values from original studies. Supplementary Figure S1: risk of bias and applicability measured by PROBAST and PRISMA Checklist.
